# Statins Versus Proprotein Convertase Subtilisin/Kexin Type 9 Inhibitors- Are We Doing Better? A Systematic Review on Treatment Disparity

**DOI:** 10.7759/cureus.10965

**Published:** 2020-10-15

**Authors:** Chetana Singh, Danel J Valero, Javariya Nisar, Jose I Trujillo Ramirez, Karisma K Kothari, Sasank Isola, Aleyda M San Hernandez, Domonick K Gordon

**Affiliations:** 1 Primary Care, California Institute of Behavioral Neurosciences & Psychology, Fairfield, USA; 2 Anesthesia, California Institute of Behavioral Neurosciences & Psychology, Fairfield, USA; 3 Internal Medicine, California Institute of Behavioral Neurosciences & Psychology, Fairfield, USA; 4 Medicine, Xavier University School of Medicine, Oranjestad, ABW; 5 Psychiatry, California Institute of Behavioral Neurosciences & Psychology, Fairfield, USA; 6 Internal Medicine, Scarborough General Hospital, Scarborough, TTO

**Keywords:** hyperlipidemia treatment, intensity of statin dosing, statin therapy, pcsk9 inhibitors, community health & primary healthcare research, healthcare inequality, health disparities and vulnerable populations, women’s health, treatment guidelines, access to healthcare

## Abstract

Coronary artery disease (CAD) is a significant contributor to mortality in America. A common risk factor of CAD is hyperlipidemia. Treatment guidelines of hyperlipidemia are well established. Statins are the cornerstone of treating hyperlipidemia. New medications such as proprotein convertase subtilisin/kexin type 9 inhibitors (PCSK9 inhibitors) have also illustrated significant results in treating hyperlipidemia. While multiple studies exemplify the disparities in statin and PCSK9 inhibitors utilization to reduce CAD mortality and risk factors, there are no systematic reviews to validate these disparities. We conducted a search on PubMed, including Medline and PubMed Central, and Google Scholar. For this analysis, we selected articles published between 2000 and 2020 and those that fit the inclusion and exclusion criteria. Based on the type of study, we performed appropriate quality assessments and deleted studies with a score of less than seven or with a high risk of biases. The search strategy resulted in 322 studies. After inclusion and exclusion criteria were applied, we included 20 articles in the analysis of this review. This systematic review demonstrates that non-white races and women were less likely to receive the correct, clinically indicated, therapy for hyperlipidemia. A multi-faceted approach is required to solve this inequality in healthcare.

## Introduction and background

One in every four deaths in America is attributed to heart disease [1]. The most common heart disease in the United States is coronary artery disease (CAD) [2]. CAD is defined as the narrowing of the heart vessels, leading to myocardial infarction (MI) and heart failure (HF). CAD's pathophysiology is initiated by endothelial damage leading to a chronic inflammatory process, followed by lipid accumulation, smooth muscle proliferation, cell death, fibrosis, and eventually narrowing of the artery walls. The accumulation of low-density lipoprotein (LDL) cholesterol and its oxidation is an important event in the formation of the lesion [3]. 

This arterial stenosis can be attributed to both environmental and genetic factors [3]. Some risk factors for CAD have already been studied and established. These include hyperlipidemia, hypertension, diabetes mellitus (DM), and smoking [3]. Moreover, factors such as gender, race, and age have illustrated to impact mortality in CAD. People >65 years old tend to have higher mortality due to CAD. Women generally present with CAD later in life and, therefore, also have higher mortality. Similarly, there is a larger disparity between white women and black women, with black women illustrating the most mortality [4]. Thus, these adversely impacted groups require aggressive risk factor modifications. 

The American Heart Association (AHA) has implemented a program called My Life Check- Life's Simple Seven to summarize cardiovascular health risk factors, including hyperlipidemia. Therefore, reducing lipid levels and recognizing who would benefit from a targeted reduction in lipid levels are cornerstones of decreasing mortality in CAD [2]. The efficacy and safety of lipid-lowering medications such as statins and proprotein convertase subtilisin/kexin type 9 inhibitors (PCSK9 inhibitors) have been thoroughly studied and proven beneficial in lowering lipid levels [5,6]. A four-fold increase in lipid-lowering medications has been noted and accounts for the decrease in CAD risk reduction in Americans between 1990 and 2010 [7,8]. 

To ensure that the right patients are targeted with lipid-lowering therapy, guidelines such as Adult Treatment Plan III (ATP III) in 2011, followed by AHA guidelines in 2013 [9], were established. Per the AHA guidelines, four groups have shown the most risk reductions due to statin therapy and, therefore, should have statin prescriptions. These groups include- A) Anyone with clinical atherosclerotic coronary vascular disease (ASCVD); B) Anyone with an intimal LDL of >190 mg/dl; C) People between the ages of 40-75 years who are diagnosed with DM and have an initial LDL of 70-189 without clinical ASCVD; D) People between the ages of 40-75 without DM and ASCVD, but with an LDL of 70-189 and an estimated 10-year ASCVD risk score of ≥7.5% [2]. 

Furthermore, the use of PCSK9 inhibitors such as evolocumab and alirocumab for treating high-risk patients and those without a reduction in LDL after statin use is well documented [10,11]. Clinical trials have provided evidence of PCKS9 inhibitors' efficacy and safety to reduce cardiovascular events [12].

This update in guidelines should lead to more people of all races and gender to be eligible for statin use. This should be beneficial for non-white people and those with lower socioeconomic backgrounds, as CAD risks are higher in these individuals [2]. However, the out of pocket costs of evolocumab poses a threat to this attainment of better outcomes. In addition, studies have illustrated that statin use is lower in patients who face multiple vulnerabilities, female sex, black race, and older adults [13-16]. Furthermore, a treatment gap in hyperlipidemia is associated with higher risks for adverse events [17]. Lastly, data from the REasons for Geographic and Racial Differences in Stroke (REGARDS) study shows that even after accounting for healthcare access, white diabetic men with LDL >100 were more likely to be prescribed statins and reach their LDL goals than black diabetic men and diabetic women of both races [18]. 

While multiple studies have exemplified the disparities in statin and PCSK9 inhibitors utilization to reduce CAD mortality and risk factors, there are no systematic reviews to validate these disparities. With the diversity in America increasing in the coming years, and the clinical and economic costs of cardiovascular events being high [19], promptly solving this problem is indispensable. The objective of this systematic review is to bring these disparities to the forefront. This study will address the race and sex disparity in treating hyperlipidemia with statins versus PCSK9 inhibitors.

## Review

Methods

The Preferred Reporting Items for Systematic Reviews and Meta-Analyses (PRISMA) checklist was utilized throughout the compilation of this systematic review to answer the research question: Is there a disparity between race and gender in hyperlipidemia treatment with statin versus PCSK9 inhibitors? We performed multiple reiterations of various searches to uncover the articles. We performed the search on databases such as PubMed, including Medline and PubMed Central (PMC), and Google Scholar. Handpicking from discovered articles was also implemented to attain desirable results. For this study, we used the following keywords: statin therapy, PCSK9 inhibitors treatment of hyperlipidemia, race disparities, gender disparities, and healthcare disparities. 

Inclusion criteria included articles published in the last two decades (2000-2020), written in English, incorporated statin or PCSK9 inhibitors as therapy for hyperlipidemia, and compared these treatments to genders and or race. Genders included male or female, and race included African American, non-Hispanic whites, and Hispanics. We excluded articles that were only abstracts, about treatments such as lifestyle change, or published outside the United States of America. 

First, we utilized PubMed to find articles regarding disparities in healthcare and hyperlipidemia treatment with statins, using boolean search terms and Medical Subject Headings (MeSH) terms (Table 1). 

**Table 1:** Search Results for Healthcare Disparities in Treatment of Hyperlipidemia With Statins. ^+^MeSH: Medical Subject Headings. ^*^PMC: PubMed Central.

Keyword- 8.4.20	Database	Studies
Statin Therapy	PubMed- Medline and PMC^*^	42,269
race disparities	PubMed- Medline and PMC	23,183
gender disparities	PubMed- Medline and PMC	18,202
((Statin Therapy) AND (race disparities)) AND (gender disparities)	PubMed- Medline and PMC	33
MeSH^+^ Keyword 8.4.20		
HYPERLIPIDEMIAS	PubMed- Medline and PMC	66,111
SEX	PubMed- Medline and PMC	7,652
("GENDER"[ MeSH])	PubMed- Medline and PMC	0
("RACE"[ MeSH])	PubMed- Medline and PMC	0
"Healthcare Disparities" [ MeSH]	PubMed- Medline and PMC	16,893
(("Hyperlipidemias"[ MeSH]) AND "Sex"[ MeSH])	PubMed- Medline and PMC	11
(("Hyperlipidemias"[ MeSH])) AND ("Healthcare Disparities"[ MeSH])	PubMed- Medline and PMC	39

Next, we implemented the following search terms "PCSK9 inhibitor therapy and disparities" in PubMed. Because of the low results yielded, the search was recreated on Google Scholar using the search terms: "PCSK9 inhibitors for hyperlipidemia and disparities in race and gender." We obtained full articles from PubMed and Google Scholar (Table 2).

**Table 2:** Search Results for Healthcare Disparities in Treatment of Hyperlipidemia With PCSK9 Inhibitors. ^+^PCSK9: Proprotein convertase subtilisin/kexin type 9. ^*^PMC: PubMed Central.

Keyword- 8.12.20	Database	Studies
PCSK9^+^ inhibitor therapy	PubMed- Medline and PMC^*^	1,527
race disparities	PubMed- Medline and PMC	23,183
gender disparities	PubMed- Medline and PMC	18,202
([PCSK9 inhibitor therapy] AND [race differences]) AND (gender differences)	PubMed- Medline and PMC	0
PCSK9 inhibitor therapy and disparities	PubMed- Medline and PMC	6
PCSK9 inhibitors for hyperlipidemia and disparities in race and gender	Google Scholar	582
PCSK9 inhibitors for hyperlipidemia and disparities in race and gender in 2000-2020	Google Scholar	244

**Figure 1:**
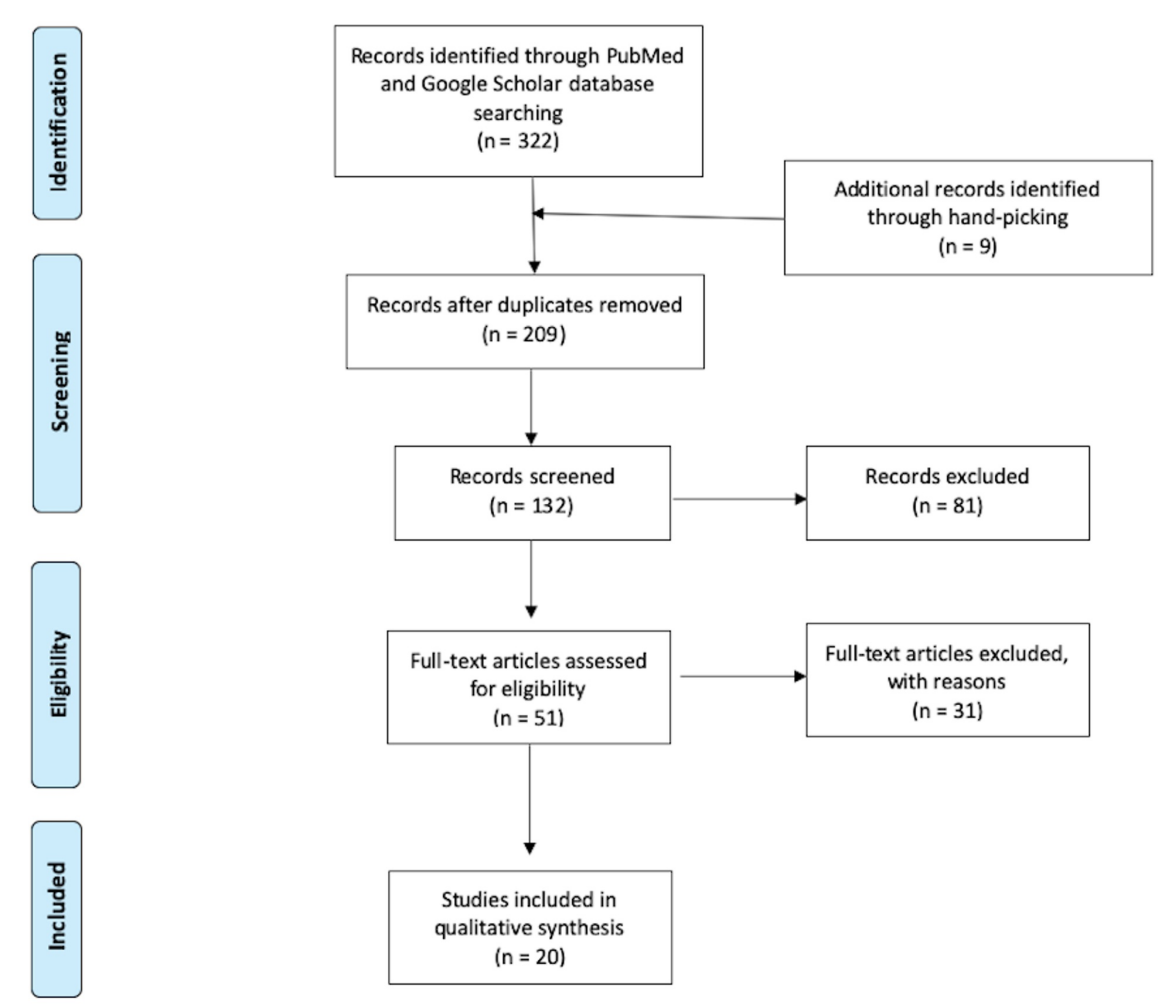
PRISMA Flow Diagram of the Search Results. PRISMA: Preferred Reporting Items for Systematic Reviews and Meta-Analyses.

Depending on the type of research articles, we used various quality assessment tools. These included- The Newcastle-Ottowa Scale for cross-sectional, case-control, and cohort studies, and the Amstar checklist to determine the quality of systematic reviews and meta-analysis. For this review, we excluded articles that scored less than 7 in the quality assessments or classified as "moderate-high risk of bias" (Figure 1). 

Results

The search strategy resulted in a total of 322 studies. We perused all the abstracts, removed all the duplicates, and that left us with 200 articles. We added nine articles through handpicking from the references of reviewed articles. We shortlisted 51 abstracts based on the inclusion and exclusion criteria, and we excluded 27 articles after a full-text-review. Lastly, we performed quality assessments, and we excluded four articles for a quality assessment score of less than seven. Therefore, we reviewed a total of 20 articles for this paper (Table 3). Due to the research question's nature, most of the studies in the review are cross-sectional, cohort studies, and one meta-analysis. The study sample was a total of 3,491,828, ranging from 1,066 to 2,560,856 subjects. 

**Table 3:** Summarizes All Studies That Were Analyzed, With Quality Assessment Tools and Quality Assessment Scores. ^+^PCSK9: Proprotein Convertase Subtilisin-Kexin Type 9, PALM: Patient and Provider Assessment of Lipid Management, NOS: Newcastle-Ottawa Scale

Title	Author	Year of Publication	Type of Study	Number of Participants	Purpose of the Study	Quality Assessment Tool	Quality Assessment Score
Patient‐Reported Reasons for Declining or Discontinuing Statin Therapy: Insights from the PALM Registry	Bradley et al. [20]	2019	Cross-Sectional	5,693	Patient-reported reasons for statin underutilization.	NOS*	7
Association of Patient Perceptions of Cardiovascular Risk and Beliefs on Statin Drugs with Racial Differences in Statin Use	Nanna et al. [21]	2018	Cross-Sectional	5,689	Cause of statin underutilization in the African American population.	NOS*	9
Vulnerabilities to Health Disparities and Statin Use in the REGARDS (Reasons for Geographic and Racial Differences in Stroke) Study	Schroff et al. [13]	2017	Cross-Sectional	18,216	Chances of being on statins with individual vulnerability and cumulative vulnerabilities. Vulnerabilities include- Black race, female sex, increasing age, low socioeconomic status, no health insurance.	NOS*	8
Quantifying sociodemographic and income disparities in medical therapy and lifestyle among symptomatic patients with suspected coronary artery disease: a cross-sectional study in North America	Ladapo et al. [17]	2017	Cross-Sectional	10,003	Potential gaps in preventative care in patients diagnosed with symptomatic coronary artery disease and dyslipidemia.	NOS*	7
Race-Sex Differences in Statin Use and Low-Density Lipoprotein Cholesterol Control Among People with Diabetes Mellitus in the Reasons for Geographic and Racial Differences in Stroke Study	Gamboa et al. [18]	2017	Cross-Sectional	2482	To study race-sex statin treatment patterns in those with diabetes.	NOS*	8
Health Disparities in Familial Hypercholesterolemia	Amrocet et al. [22]	2017	Cross-Sectional	3167	To study race and sex disparities for treatment with a statin in patients from the CAscade SCreening for Awareness and DEtection of Familial Hypercholesterolemia (CASCADE-FH) registry- a national registry for familial hypercholesterolemia.	NOS*	9
Underuse of Effective Cardiac Medications Among Women, Middle-Aged Adults, and Racial/Ethnic Minorities with Coronary Artery Disease (from the National Health and Nutrition Examination Survey 2005 to 2014)	Tran et al. [23]	2017	Cross-Sectional	1,789	Describe decade long trends in adults with coronary artery disease based on age, sex, and race.	NOS*	7
Antiplatelet and Statin Use in US Patients with Coronary Artery Disease Categorized by Race/Ethnicity and Gender, 2003 to 2012	Johansen et al. [24]	2015	Cross-Sectional	14,334	Race and ethnic disparities in treatment with a statin in patients with known coronary heart disease.	NOS*	7
Gender and racial disparities in adherence to statin therapy: a meta-analysis	Lewey et al. [25]	2013	Meta-Analysis	2,560,856	Illustrate the effect of race and gender on adherence to a statin for primary or secondary prevention.	Amstar	Low risk of Bias
A prospective study of statin use and mortality among 67,385 blacks and whites in the Southeastern United States	Lipworth et al. [8]	2013	Cross-Sectional	67,385	Statin use in different races and their effects on mortality.	NOS*	9
Racial and ethnic disparities in cardiovascular medication use among older adults in the United States	Qato et al. [15]	2013	Cross-Sectional	1066	Disparities in the use of statins in subjects with known cardiovascular disease risk.	NOS*	8
Cholesterol treatment with statins: who is left out, and who makes it to goal?	Franks et al. [14]	2010	Cross-Sectional	18,042	If sociodemographic factors are associated with failure to be on statin therapy and the inability to be at low-density lipoprotein goal.	NOS*	7
Effect of Access to Prescribed PCSK9^+^ Inhibitors on Cardiovascular Outcomes	Myers et al. [26]	2019	Retrospective Cohort Study	139,036	To illustrate death and complications due to coronary heart disease are higher in those whose PCKS9 inhibitors prescriptions were denied.	NOS*	8
Trends and Factors Associated With Insurer Approval of Proprotein Convertase Subtilisin/Kexin Type 9 Inhibitor Prescriptions	Doshi et al. [27]	2019	Cross-Sectional	12,309	To examine insurer approval rates for PCSK9 inhibitors and reasons for rejection.	NOS*	7
National Trends in Nonstatin Use and Expenditures Among the US Adult Population From 2002 to 2013: Insights From Medical Expenditure Panel Survey	Salami et al. [28]	2018	Cohort Study	157,719	To demonstrate the utilization and expenditure of nonstatin use (includes PCSK9 inhibitors).	NOS*	10
Prescribing Patterns of Proprotein Convertase Subtilisin-Kexin Type 9 Inhibitors in Eligible Patients With Clinical Atherosclerotic Cardiovascular Disease or Heterozygous Familial Hypercholesterolemia	Karalis et al. [29]	2018	Retrospective Cohort Study	368,624	To compare and illustrate the differences between those who were prescribed PCSK9 inhibitors vs. those that were eligible and not prescribed.	NOS*	8
Variation in Lipid-Lowering Therapy Use in Patients with LDL-C ≥ 190mg/dL: Insights from the NCDR®PINNACLE Registry	Virani et al. [30]	2018	Cross-Sectional	49,447	To demonstrate the variation in lipid-lowering therapy (including PCSK9 inhibitor) in patients with low-density lipoprotein ≥190 mg/dl in a national sample.	NOS*	8
Patient characteristics and real-world treatment patterns among early users of PCSK9 inhibitors	Rane et al. [31]	2018	Cross-Sectional	1,522	To differentiate between patients who were prescribed PCSK9i and those who were not, and to characterize these patients.	NOS*	7
Association of Prior Authorization and Out-of-pocket Costs With Patient Access to PCSK9 Inhibitor Therapy	Navar et al. [32]	2017	Cross-Sectional	45,092	To evaluate factors affecting patient access to newly prescribed PCSK9 inhibitors.	NOS*	8
Proprotein Convertase Subtilisin/Kexin Type 9 Inhibitor Therapy: Payer Approvals and Rejections, and Patient Characteristics for Successful Prescribing	Heset et al. [33]	2017	Retrospective Cohort Study	9,357	To assess factors associated with acceptance and rejection of PCSK9 inhibitors prescription claims.	NOS*	7

Discussion

Treatment gaps in patients with hyperlipidemia are discernable and well established in the scientific literature. Gaps exist with all treatment therapies, including the utilization of both statin and PCSK9 inhibitors. Our systematic review illustrated that these differences are still rampant today. 

Statin and Healthcare Disparities

Statins' efficacy and safety are equivocal in both men and women [5]. Statin is a cornerstone therapy in the treatment of hyperlipidemia in both primary and secondary prevention. Yet, there is a large disparity between those receiving statins and those that are not. Per Bradley et al., 28% of the qualifying population were not taking statins, and the majority (58%) were not prescribed statins by their physicians. The patient demographics who were never prescribed statins included African Americans, women, and those without insurance. Perceived side effects such as muscle aches, liver failure, and memory loss were the primary reason for patients on statins to stop. Nevertheless, the willingness to try statins again, despite perceived side effects, was high. Additionally, the readiness to take statins, if not previously prescribed, was also high in this population. Therefore, better educational material for physicians describing those who should be prescribed statins and their side effects would be beneficial. In addition, studies have found that statin utilization is low in the general population [21]. However, statin use continues to be lower in the African American community. The differences in perception between the two races cane explain these variances. Primarily, African Americans did not think that statins were necessary, nor did they think they were in a higher cardiovascular risk category. Furthermore, fewer African Americans reported complete trust in their physicians. Thus, better communication tactics and increased trust-building would benefit the African American community and decrease treatment gaps. 

The lack of insurance proved to be a prominent architect of disparity in attaining treatment with statins. Additionally, the disparities in treatment with statins increased with an accumulation of vulnerabilities, such as older age, female sex, African American race, increase poverty, and lack of insurance. The most striking statistic of this study was that black women comprised 90% of the group with all five vulnerabilities, even though they only formed 27% of the study sample [13]. Similarly, variation in hyperlipidemia treatment is statistically significant, with women and older patients being significantly less likely to be on statin therapy, which reportedly led to adverse events [17]. Patient and clinician education to illustrate the benefits of primary prevention of hyperlipidemia are necessary. Physicians prescribing trends are a well-documented instigator of treatment disparity as it is a subjective model. For instance, Gamboa et al. postulate that physicians presumed white men to have a higher risk than other race and sex groups and, therefore, treated them more aggressively [18]. Thus, new guidelines state that risk factor calculators are used at point-of-care to move towards a more objective treatment method. 

In contrast, although the use of statins has increased within the study period, its use is still low amongst women and ethnic minorities diagnosed with CAD [23]. Moreover, when controlled for sociodemographic factors, African Americans and Hispanic men and all races of women had significantly lower rates of being on statins [24]. Therefore, while insurance and lack of access might be factors of the treatment gap, it does not entirely account for it. One reason could be adherence to treatment with statins. Lewey et al. exemplified that women and non-white races were more like to be nonadherent to statins due to adverse events or mistrust in the healthcare system [25]. Thus, patient education on the treatment, its adverse events, and the healthcare system could alleviate the gap. 

To juxtapose, Lipworth et al. garnered their study population from similar socioeconomic backgrounds, and they demonstrated that African Americans revealed a low self-reported prevalence of hypercholesterolemia when compared to their white counterparts [8]. Thus, there is a need for more public outreach and education to the minority races to increase their awareness of hyperlipidemia and its treatment options. The study did continue to elucidate a modest difference between treatment for high cholesterol with statins between whites and African Americans; however, undertreatment in African American men and women was still prevalent. Also, they demonstrated that using statins led to a decrease in mortality in both men and women. Franks et al. demonstrated that older men with low socioeconomic backgrounds were significantly less likely to be on statins and attain LDL goals due to statin out-of-pocket costs and formulary restrictions. Considering this study was published in 2010, and Lipitor's patent ended in 2011- allowing the advent of generic atorvastatin, this statistic is bound to have changed in recent years. Moreover, there is a difference in the diffusion of new treatment and technologies in low socioeconomic communities, which intensifies the treatment gaps [14]. 

To compare, Qato et al. demonstrated that while less than half of a high-risk population were on statins, older African American males were less likely to be prescribed statin, and this was after adjusting for socioeconomic barriers and access to care difficulties [15]. Therefore, there needs to be an improvement in prescription and adherence. 

In conclusion, non-white races, women, and older age were all associated with fewer statin prescriptions to treat hyperlipidemia. Physician education regarding the current guidelines for hyperlipidemia, better communication tactics between physicians and the marginalized population will increase trust between these communities and help offset this imbalance. Additionally, patient education about the importance of hyperlipidemia treatment and statins' expected side effects is essential to decrease the treatment gap. 

PCSK9 Inhibitors and Health Disparities

In 2015 the Food and Drug Administration approved two new drugs - alirocumab and evolocumab, both PCSK9 inhibitors. They were approved for lowering LDL levels in patients with familial hyperlipidemia and those with high ASCVD risks [34]. Multiple studies were performed on these drugs to illustrate their effectiveness in both men and women [6]. However, these life-altering drugs are still limited in use because of their novelty and difficulty in accessing these medications. The inability to access proper care, such as PCSK9 inhibitors, has been associated with increased cardiovascular events [26]. Insurers have set up administrative obstacles such as formulary exclusions and prior authorizations (PA) to outset the high costs. This update resulted in an increase in insurance rejections of PCSK9 inhibitors, initially due to formulary coverage and then due to PA forms [27]. By 2017, at the end of the study period, only two in five eligible patients were approved for PCSK9 inhibitors. Thus, patients with limited access to healthcare would never be prescribed, nor will they be able to afford these medications [27]. As such, while costs and expenditures on such non-statin lipid-lowering therapies have increased four-fold- women, the uninsured, African Americans, and Hispanics were less likely to be on these medications [28]. Similarly, Karalis et al. illustrated that only 0.5% out of all eligible patients were prescribed PCSK9 inhibitors; and although 41,068 African American patients were eligible to be on PCSK9 inhibitors, only 89 (0.22%) were prescribed this. On the other hand, out of 310,666 white patients that were eligible to be on PCSK9 inhibitors, only 1,602 (0.52%) were prescribed [29]. This confirms the actuality of discrepancies in prescribing PCSK9 inhibitors. Thus, even without considering insurance companies, physicians need to learn the guidelines of PCSK9 inhibitors prescription.

Undertreatment and incorrect treatment with high variations in care of high LDL are shared among all sex-race groups [30]. While both ATP III and ACC guidelines recommend calculating the ASCVD risk scores to treat patients within the proper guidelines, only 48% of the physicians perform risk score calculations in patients with LDL ≥ 190mg/dl. Physicians might not corroborate with the guidelines on what is high risk [30]. In contrast, Rane et al. found that physicians were prescribing PCSK9 inhibitors correctly to those with high ASCVD risks [31]. Even with the physicians correctly prescribing PCSK9 inhibitors and equally amongst male and female sex, approval rates varied amongst pharmacies, and patients would abandon their prescriptions due to co-pay costs [32]. However, patients with prescriptions from primary care physicians did not get their prescriptions approved [33]. Therefore, non-primary care physicians and cardiologists were required to prescribe lipid-lowering therapy of PCSK9 inhibitors [34]. This deepens the barrier of access to care for low socioeconomic patients and those without insurance.

The lack of insurance leads to worse clinical outcomes [35]. This persists in patients prescribed PCSK9 inhibitors due to the high costs. Even with insurance coverage, such as Medicare, the approval rates for PCSK9 inhibitors are relatively low due to PA form complications. In addition, high variation in PCSK9 inhibitors treatment is present. Therefore, physician education on guidelines for treatment with PCSK9 inhibitors, and how to navigate the administrative obstacles of PA forms, will ensure that all clincally required patients are appropriately on these medications. 

Limitations

This systematic review lacks higher evidence studies, such as randomized control trials. Due to the nature of the research question, we uncovered more observational studies. Observational studies, by definition, are prone to selection bias due to a lack of randomization. Likewise, observational studies can only guarantee associations and not causation. However, without inherent biases at work, associations between race and sex and treatment of hyperlipidemia would not exist. The large sample size of this study, nevertheless, is highly favorable. Self-report was the method of choice to gather data for most of the studies, which is sensitive to recall biases. Missing data were also noted in some studies; yet, this was controlled. Furthermore, while "race" was used as a generalized term, studies involving Hispanic and Asian populations were few. In the future, more studies, including these populations and the female sex, will be pivotal for the growing population of America.

## Conclusions

This systematic review examined the race and sex incongruences in the treatment of hyperlipidemia. Hyperlipidemia is a known risk factor for CAD and can be treated with statins and PCSK9 inhibitors. Though multiple guidelines have been established to ensure a uniform treatment of high LDL throughout the population, their use has not been pervasive. This analysis illustrated that African American men and women of all races were less likely to receive statin therapy. Similar results were found with those being treated with PCSK9 inhibitors. Insurance rejections, high copayments, physician perception of high risk, and African Americans’ lack of trust in the healthcare system all played a colossal part in the discrepancy in using statins or PCSK9 inhibitors to treat hyperlipidemia.

A multi-pronged approach to solve this disparity would be necessary. A decrease in drug prices, as seen with the availability of generic Lipitor, might be pivotal in improving medication adherence. However, a better community outreach program, targeting women and African Americans is necessary to improve medication adherence and to articulate the risk associated with high LDL levels. Educational outreach to the physicians regarding communication skills, building trust, and up to date guidelines are also imperative. Even with limitations, this systematic review concisely demonstrated that race and sex imparity in the treatment of hyperlipidemia persists. Moreover, this review has illustrated the important approaches to reduce this treatment gap. 

## References

[1] Heron M (2020). Deaths: leading causes for 2017. Natl Vital Stat Rep.

[2] Benjamin EJ, Muntner P, Alonso A (2019). Heart disease and stroke statistics-2019 update: a report from the American Heart Association. Circulation.

[3] Singh RB, Mengi SA, Xu YJ, Arneja AS, Dhalla NS (2020). Pathogenesis of atherosclerosis: a multifactorial process. Exp Clin Cardiol.

[4] Ferdinand KC (2020). Ethnic, gender, and age-related differences in the treatment of dyslipidemia. Am J Manag Care.

[5] Cholesterol Treatment Trialists' (CTT) Collaboration, Fulcher J, O'Connell R (2015). Efficacy and safety of ldl-lowering therapy among men and women: meta-analysis of individual data from 174,000 participants in 27 randomised trials. Lancet.

[6] Ganda O (2018). Beyond statins: who and when to prescribe?. Curr Diab Rep.

[7] Mitchell UA, Ailshire JA, Kim JK, Crimmins EM (2019). Black-White differences in 20-year trends in cardiovascular risk in the United States, 1990-2010. Ethn Dis.

[8] Lipworth L, Fazio S, Kabagambe EK (2014). A prospective study of statin use and mortality among 67,385 blacks and whites in the southeastern united states. Clin Epidemiol.

[9] (2020). National cholesterol education program: ATP III guidelines at-a-glance quick desk reference. https://www.nhlbi.nih.gov/files/docs/guidelines/atglance.pdf.

[10] Schmidt AF, Pearce LS, Wilkins JT, Overington JP, Hingorani AD, Casas JP (2017). PCSK9 monoclonal antibodies for the primary and secondary prevention of cardiovascular disease. Cochrane Database Syst Rev.

[11] Nicholls SJ, Puri R, Anderson T (2016). Effect of evolocumab on progression of coronary disease in statin-treated patients: the GLAGOV randomized clinical trial. JAMA.

[12] Rosenson RS, Hegele RA, Koenig W (2019). Cholesterol-lowering agents PCSK9 inhibitors today and tomorrow. Circ Res.

[13] Schroff P, Gamboa CM, Durant RW, Oikeh A, Richman JS, Safford MM (2017). Vulnerabilities to health disparities and statin use in the REGARDS (Reasons for Geographic and Racial Differences in Stroke) Study. J Am Heart Assoc.

[14] Franks P, Tancredi D, Winters P, Fiscella K (2010). Cholesterol treatment with statins: who is left out and who makes it to goal?. BMC Health Serv Res.

[15] Qato DM, Lindau ST, Conti RM, Schumm LP, Alexander GC (2010). Racial and ethnic disparities in cardiovascular medication use among older adults in the United States. Pharmacoepidemiol Drug Saf.

[16] Robinson JG, Booth B (2010). Statin use and lipid levels in older adults: national health and nutrition examination survey, 2001 to 2006. J Clin Lipidol.

[17] Ladapo JA, Coles A, Dolor RJ (2017). Quantifying sociodemographic and income disparities in medical therapy and lifestyle among symptomatic patients with suspected coronary artery disease: a cross-sectional study in North America. BMJ Open.

[18] Gamboa CM, Colantonio LD, Brown TM, Carson AP, Safford MM (2017). Race-Sex differences in statin use and low-density lipoprotein cholesterol control among people with diabetes mellitus in the reasons for geographic and racial differences in stroke study. J Am Heart Assoc.

[19] Fox KM, Wang L, Gandra SR, Quek RGW, Li L, Baser O (2016). Clinical and economic burden associated with cardiovascular events among patients with hyperlipidemia: a retrospective cohort study. BMC Cardiovasc Disord.

[20] Bradley CK, Wang TY, Li S (2019). Patient-Reported reasons for declining or discontinuing statin therapy: insights from the PALM registry. J Am Heart Assoc.

[21] Nanna MG, Navar AM, Zakroysky P (2018). Association of patient perceptions of cardiovascular risk and beliefs on statin drugs with racial differences in statin use: insights from the patient and provider assessment of lipid management registry. JAMA Cardiol.

[22] Amrock SM, Duell PB, Knickelbine T (2017). Health disparities among adult patients with a phenotypic diagnosis of familial hypercholesterolemia in the CASCADE-FH™ patient registry. Atherosclerosis.

[23] Tran HV, Waring ME, McManus DD, Erskine N, Do VTH, Kiefe CI, Goldberg RJ (2017). Underuse of effective cardiac medications among women, middle-aged adults, and racial/ethnic minorities with coronary artery disease (from the National Health and Nutrition Examination Survey 2005 to 2014). Am J Cardiol.

[24] Johansen ME, Hefner JL, Foraker RE (2015). Antiplatelet and statin use in us patients with coronary artery disease categorized by race/ethnicity and gender, 2003 to 2012. Am J Cardiol.

[25] Lewey J, Shrank WH, Bowry AD, Kilabuk E, Brennan TA, Choudhry NK (2013). Gender and racial disparities in adherence to statin therapy: a meta- analysis. Am Heart J.

[26] Myers KD, Farboodi N, Mwamburi M (2019). Effect of access to prescribed PCSK9 inhibitors on cardiovascular outcomes. Circ Cardiovasc Qual Outcomes.

[27] Doshi JA, Li P, Puckett JT, Pettit AR, Raman S, Parmacek MS, Rader DJ (2020). Trends and factors associated with insurer approval of proprotein convertase subtilisin/kexin type 9 inhibitor prescriptions. Value Health.

[28] Salami JA, Warraich HJ, Valero-Elizondo J (2018). National trends in nonstatin use and expenditures among the US adult population from 2002 to 2013: insights from medical expenditure panel survey. J Am Heart Assoc.

[29] Karalis DG, Mallya UG, Ghannam AF, Elassal J, Gupta R, Boklage SH (2018). Prescribing patterns of proprotein convertase subtilisin-kexin type 9 inhibitors in eligible patients with clinical atherosclerotic cardiovascular disease or heterozygous familial hypercholesterolemia. Am J Cardiol.

[30] Virani SS, Kennedy KF, Akeroyd JM (2018). Variation in lipid-lowering therapy use in patients with low-density lipoprotein cholesterol >/=190 mg/dl: insights from the national cardiovascular data registry-practice innovation and clinical excellence registry. Circ Cardiovasc Qual Outcomes.

[31] Rane PB, Patel J, Harrison DJ (2018). Patient characteristics and real-world treatment patterns among early users of PCSK9 inhibitors. Am J Cardiovasc Drugs.

[32] Navar AM, Taylor B, Mulder H (2017). Association of prior authorization and out-of-pocket costs with patient access to PCSK9 inhibitor therapy. JAMA Cardiol.

[33] Hess GP, Natarajan P, Faridi KF, Fievitz A, Valsdottir L, Yeh RW (2017). Proprotein convertase subtilisin/kexin type 9 inhibitor therapy: payer approvals and rejections, and patient characteristics for successful prescribing. Circulation.

[34] Baum SJ, Toth PP, Underberg JA, Jellinger P, Ross J, Wilemon K (2017). PCSK9 inhibitor access barriers-issues and recommendations: improving the access process for patients, clinicians and payers. Clin Cardiol.

[35] Kesselheim AS, Huybrechts KF, Choudhry NK, Fulchino LA, Isaman DL, Kowal MK, Brennan TA (2015). Prescription drug insurance coverage and patient health outcomes: a systematic review. Am J Public Health.

